# Emergence of Tetracycline Resistant* Vibrio cholerae* O1 Biotype El Tor Serotype Ogawa with Classical* ctxB* Gene from a Cholera Outbreak in Odisha, Eastern India

**DOI:** 10.1155/2016/1695410

**Published:** 2016-01-03

**Authors:** M. Jain, P. Kumar, A. K. Goel

**Affiliations:** Biotechnology Division, Defence Research & Development Establishment, Jhansi Road, Gwalior 474002, India

## Abstract

In September 2010, a cholera outbreak was reported from Odisha, Eastern India.* V. cholerae* isolated from the clinical samples were biochemically and serologically confirmed as serogroup O1, biotype El Tor, and serotype Ogawa. Multiplex PCR screening revealed the presence of various genes, namely,* ompW*,* ctxB*,* zot*,* rfbO1*,* tcp*,* ace*,* hlyA*,* ompU*,* rtx,* and* toxR,* in all of the isolates. The isolates were resistant to co-trimoxazole, nalidixic acid, polymyxin B, spectinomycin, streptomycin, sulfamethoxazole, tetracycline, trimethoprim, and vibriostatic agent 2,4-diamino-6,7-diisopropylpteridine (O/129). Minimum inhibitory concentration of tetracycline decreased in the presence of carbonyl cyanide* m*-chlorophenylhydrazone (CCCP), suggesting the involvement of efflux pumps. PCR analysis confirmed the presence of class I integrons as well as SXT elements harbouring antibiotic resistance genes in all isolates. Sequencing revealed the presence of* ctxB* gene of classical biotype in all the isolates. The isolates harboured an RS1-CTX prophage array with El Tor type* rstR *and classical* ctxB *on the large chromosome. The study indicated that the* V. cholerae* El Tor variants are evolving in the area with better antibiotic resistance and virulence potential.

## 1. Introduction 

Cholera is still a major health problem of global importance as evidenced by the recent outbreaks in Haiti and Zimbabwe. In 2013, a total of 47 countries from all continents reported 129 064 cases of cholera including 2102 deaths with a case fatality rate of 1.63% [1]. Toxigenic* Vibrio cholerae, *the causative agent of cholera, has exhibited several changing patterns in its biotype as well as in drug resistance for its better survival and infection [2, 3]. Classical biotype* V. cholerae* strains after causing earlier six cholera pandemics were replaced by El Tor biotype strains in the seventh pandemic. The variant of* V. cholerae* El Tor strain possessing cholera toxin of classical biotype appeared after the emergence of O139 strain and has now become prevalent worldwide [4]. Antimicrobial susceptibility pattern has continuously changed for* V. cholerae *[2]. Tetracycline is an important broad spectrum antibiotic used for the prophylaxis and treatment of variety of bacterial infections including listed biothreat agents* Bacillus anthracis*,* Francisella tularensis*, and* Yersinia pestis *[5]. It has been a preferred choice for control of cholera as recommended by WHO [6]. Earlier, we reported a novel mutation in* ctxB* gene of outbreak isolates in 2007 from Rayagada (19.09°N 83.27°E), Odisha, and later on the same allelic variation was observed in the Haitian outbreak isolates [7, 8]. These isolates were multidrug resistant but sensitive to tetracycline [8]. In September-October, 2010, another cholera outbreak was observed in the same area of Rayagada, Odisha. In this communication, we report the emergence of tetracycline resistant* V. cholerae* from this outbreak of Eastern India.

## 2. Materials and Methods

### 2.1. Isolation and Identification of* V. cholerae*

A total of 27* V. cholerae* isolates were recovered from randomly selected patients during the cholera outbreak in the month of September, 2010, from Rayagada district of Odisha (19.09°N 83.27°E). Samples were collected using sterile rectal swabs from the patients and processed as described earlier [9].* V. cholerae* isolates were identified and confirmed by using standard biochemical methods. Serological identification of the isolates was done by slide agglutination using commercially available polyvalent antiserum against* V. cholerae* O1 (Difco, USA). Voges Proskauer (VP) test, sheep erythrocyte haemolysis, and polymyxin B susceptibility tests were performed for determination of biotype of isolates. Other bacterial reference strains used in the study were* V. cholerae* O1 El Tor (ATCC 14033),* V. cholerae* O1 classical (ATCC 11623), and* V. cholerae* O139 (ATCC 51394).

### 2.2. PCR Assays for Virulence and Species-Specific Genes

Genomic DNA was extracted from each of the isolates using genomic DNA purification kit (MBI Fermentas, Vilnius, Lithuania) and screened for the presence of diverse gene traits by two sets of multiplex PCR (mPCR) as described elsewhere [10]. The first mPCR detected the presence of genes encoding the outer-membrane protein (*ompW*), cholera toxin (*ctxB*), zonula occludens toxin (*zot*), O1 somatic antigen (*rfbO1*), and toxin coregulated pilus (*tcp*). The second mPCR amplified other virulence and toxigenic genes encoding the accessory cholera enterotoxin (*ace*), haemolysin (*hlyA*), outer-membrane protein (*ompU*), repeat in toxin protein (*rtx*), and toxin regulator (*toxR*).

### 2.3. Antimicrobial Drug-Susceptibility Testing

The antimicrobial susceptibility of the* V. cholerae* isolates was determined by the Kirby-Bauer disc diffusion method on Mueller-Hinton agar [11], and minimum inhibitory concentration (MIC) was calculated by broth dilution method according to the guidelines of Clinical Laboratory Standards Institute [12]. The antibiotic discs and antibiotic powder were procured from Oxoid limited, UK, and Sigma Aldrich, USA, respectively. The antibiotic discs used were ampicillin (10 *μ*g), chloramphenicol (30 *μ*g), ciprofloxacin (5 *μ*g), co-trimoxazole (25 *μ*g), erythromycin (10 *μ*g), nalidixic acid (30 *μ*g), polymyxin B (50 *μ*g), rifampicin (5 *μ*g), spectinomycin (100 *μ*g), streptomycin (10 *μ*g), sulphamethoxazole (100 *μ*g), tetracycline (30 *μ*g), trimethoprim (5 *μ*g), and vibriostatic agent 2,4-diamino-6,7-diisopropylpteridine, O/129 (150 *μ*g). The MIC for selected antibiotics was calculated in the presence of carbonyl cyanide* m*-chlorophenylhydrazone (CCCP) at subinhibitory concentration. Reference strains of* Escherichia coli* ATCC 25922,* Staphylococcus aureus* ATCC 25923, and* Pseudomonas aeruginosa* ATCC 27853 were used in each test for internal quality control.

### 2.4. Mobile Genetic Elements and Their Gene Cassettes

The presence of class 1 integron and SXT constins was detected by PCR as described by Hochhut et al. [13]. The presence of gene cassettes for* aadA2 *(encoding resistance for streptomycin and spectinomycin),* strA *(encoding resistance for streptomycin),* blaP1 (*encoding resistance for *β*-lactams),* floR* (encoding resistance for chloramphenicol), and* dfrA1* (encoding resistance for trimethoprim) was determined by PCR as described earlier [14, 15].

### 2.5. Sequencing of the* ctxB* Gene

The* ctxB* gene was amplified from the isolates using primers described previously [16]. Sequencing was carried out using the same PCR primers on a 96-capillary model 3730xl system using the Big Dye Terminator kit from Applied Biosystems (Applied Biosystems, Foster City, CA, USA). The sequences were edited with SeqED program (Applied Biosystems) and were aligned with sequences of* ctxB* gene of reference strains of* V. cholerae* O1 El Tor, classical, and O139. The deduced amino acid sequences of the* ctxB* gene from all the strains were aligned using CLUSTAL W.

### 2.6. Analysis of RS1 and CTX Prophage Array

Arrangement of RS1 and CTX phage was determined by PCR. Presence of the RS1 element was confirmed by PCR using the* rstC *specific primers as described elsewhere [17]. The primers for *rstR* ^El  Tor^, *rstR* ^Classical^, and *rstR* ^Calcutta^ were used for* rstR *typing of RS element and for differentiation of classical and El Tor biotype of* V. cholerae* [18]. The location of an RS1-CTX prophage array on the large chromosome, insertion of the CTX prophage on the small chromosome, and tandem repeats of RS1 and CTX prophage on chromosome were determined by PCR as described earlier [17].

## 3. Results

### 3.1. Identification of Outbreak Strains

A total of 27 clinical strains of* V. cholerae* were isolated from the affected patients during cholera outbreak in Odisha, Eastern India. On the basis of biochemical tests, all the isolates were found to belong to El Tor biotype as the isolates were VP test positive, hemolytic on sheep blood agar, and resistant to polymyxin B.

### 3.2. Gene Traits

Two sets of multiplex PCR revealed that all the outbreak isolates were invariably positive for various virulent and species-specific genes, namely,* ace *(309 bp)*, ctxAB *(536 bp)*, hlyA *(480 bp),* ompU *(655 bp)*, ompW *(304 bp)*, rfbO1 *(638 bp)*, rtxC *(265 bp)*, tcpA *(805 bp)*, toxR *(779 bp), and* zot *(947 bp) genes (Table 1).

### 3.3. Antibiotic Drug Resistance Pattern

The antimicrobial disc diffusion test revealed that all the isolates were resistant to co-trimoxazole, nalidixic acid, polymyxin B, spectinomycin, streptomycin, sulfamethoxazole, tetracycline, trimethoprim, and vibriostatic agent 2,4-diamino-6,7-diisopropylpteridine (O/129). However, the isolates were sensitive to ampicillin, chloramphenicol, erythromycin, ciprofloxacin, and rifampicin. The MIC for selected antibiotics is given in Table 2. The presence of carbonyl cyanide* m*-chlorophenylhydrazone (CCCP), an inhibitor of major facilitator superfamily (MFS) proteins in broth at subinhibitory concentration (0.5 *μ*g/mL), reduced MIC for tetracycline to eightfold (16 to 2 *μ*g/mL) and the isolates became susceptible. CCCP also reduced the MIC for trimethoprim from 1024 *μ*g/mL to 256 *μ*g/mL, whereas MIC for nalidixic acid, streptomycin, sulfamethoxazole, and ampicillin remained unaffected (Table 2).

### 3.4. Prevalence of Multidrug Resistant Elements

PCR analysis revealed the presence of class I integron as well as SXT element harbouring antibiotic resistance traits against multiple drugs in all the isolates. The isolates were positive for* aadA2 *(streptomycin and spectinomycin) and* dfrA1 *(trimethoprim) gene cassettes but negative for* blaP1 *(*β*-lactam antibiotics) cassettes [14]. The strains were also positive for* strA* carried on SXT constin that confers resistance for streptomycin. However, all the isolates were negative for* floR *(chloramphenicol) genes.

### 3.5. Sequence Analysis of* ctxB*

**Figure 1:**
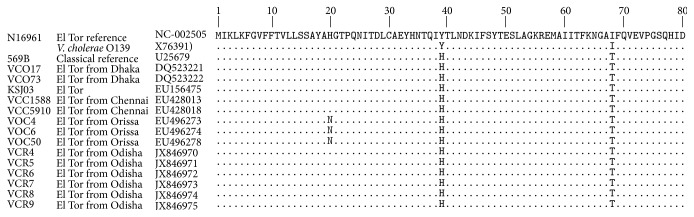
Amino acid sequence alignment of CTB subunit of* V. cholerae* O1 El Tor isolates from Odisha outbreak with preexisting reference El Tor and classical strains. Identical amino acid residues are indicated by dots. Amino acid sequences of* V. cholerae* CTB used in alignment were taken from GenBank.

The deduced amino acid sequence analysis of CT-B from the representative isolates varied from CT-B of reference El Tor strain N16961 and O139 strain at position 39 (histidine in place of tyrosine) and position 68 (threonine in place of isoleucine) but was similar to CT-B subunit of reference classical strains 569B (Figure 1). However, no mutation was observed at position 20, which was found in the* V. cholerae* isolates from the previous outbreak in 2007 (Figure 1).

### 3.6. Phage Array

**Figure 2:**
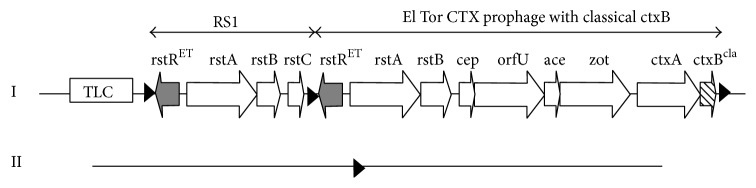
Genetic structures and CTX prophage array of* V. cholerae* strains. Block arrows indicate the transcription direction of each gene. Black triangles on the genome indicate the repeat sequence flanking the integrated phage DNA.* V. cholerae *isolates in this study contain a single RS1 element and a single classical CTX prophage after the TLC element on chromosome I.

The cholera toxin gene* ctxAB* lies on the single stranded DNA of CTX phage which is a filamentous phage [20]. There is specific site for integration of CTX phage DNA into the* V. cholerae* chromosome. Toxigenic strains contain a genetic element RS1 which is related to CTX phage. RS1 element is found adjacent (5′ or 3′) to CTX prophage. Several types of CTX phage/RS1 arrays have been found in the epidemic* V. cholerae* strains [21]. In this study,* rstC* specific primer set* rstCF*/*rstCR *amplified the DNA, indicating the presence of the RS1 element in the chromosome. A PCR product was amplified with* rstCF*/*cepR* showing the existence of RS1-CTX prophage array. In contrast,* ctxBF* and* rstCR* primer pair did not give any amplification, indicating the absence of CTX prophage-RS1 array. No DNA fragment was amplified using the primer pairs of* ctxBF/cepR* and* rstCF4/rstCR4*, indicating the absence of tandem repeats of CTX prophage and RS1 element on the genome, respectively. Location of RS1-CTX prophage array on the large chromosome was confirmed with the PCR amplified products using* Ch1F*/*rstAR *and* ctxBF*/*Ch1R *primers. The isolates yielded PCR products using* Ch2F*/*Ch2R *primers indicating the absence of the CTX prophage or RS1 element on small chromosome. The RS1-CTX prophage array has been shown in Figure 2.

## 4. Discussion

*V. cholerae *O1 El Tor strains are supposed to have better adaptability in the environment and colonize more efficiently in the intestinal lumen as compared to the classical one [22]. However, cholera toxin of classical type causes greater fluid accumulation in the intestinal lumen as compared to toxin of El Tor type [23]. Therefore, hybrid biotype property of* V. cholerae* enhances the infection potential and adaptability in the environment showing their epidemiological successfulness. Outbreaks of such variant type strains are associated with more fluid loss and increased case fatality rate [24]. It has now become a global threat as most of the outbreak strains are associated with variant El Tor strains in India and other countries [3, 7, 16].

All isolates from this outbreak were identified as* V. cholerae* O1 biotype El Tor serotype Ogawa. The isolates harboured various toxigenic and pathogenic genes (Table 1). The presence of* ompW, ctxB,* and* rfbO1* genes confirmed the* V. cholerae* species, its toxigenicity, and group 1 antigen of the “O” side chain of the LPS (serogroup), respectively. Presence of* rtxA* or* rtxC* genes determines the biotypes of* V. cholerae *O1 [25, 26]. All isolates in this study belonged to the El Tor biotype on the basis of the repeat in toxin gene (*rtxC*).

Sequencing of* ctxB* gene revealed that all* V. cholerae* O1 El Tor isolates in this study had* ctxB *sequence of the classical biotype. Classical biotype strains were replaced by the El Tor biotype in the seventh and current pandemic of cholera. Both biotypes of* V. cholerae *O1 are closely related in their O-antigen biosynthetic genes. However, the genomic structure of the CTXΦ, in which the cholera toxin genes are contained, differs between the classical and El Tor biotypes [20, 27]. On the basis of genetic structures of the CTX prophage and RS1 element on each chromosome, atypical El tor strains have been classified into two different groups [17, 28]. Group 1 strains harbor a tandem repeat of the classical CTX prophage on the small chromosome and Group II strains contain the RS1 and CTX prophage with El Tor type* rstR* and classical* ctxB* on the large chromosome [28, 29]. All the strains used in this study belonged to Group II and contained the RS1 and CTX prophage with El Tor type* rstR *and classical* ctxB *on the large chromosome.

Odisha is situated at the coast of Bay of Bengal and in general outbreaks in the state are linked to flooding and cyclones [10, 30]. The variant El Tor strains are circulating in Odisha since 2000 and appeared in Rayagada district in 2002 but the first outbreak due to this strain was recorded in 2007 [10, 31]. To the best of our knowledge, this was the first time when tetracycline resistant clone of variant El Tor strain caused an outbreak in Odisha. Recently, tetracycline resistant* V. cholerae* strains have been reported from several other parts of India [32, 33]. Tetracycline has been a drug of choice for control of severe cases of cholera [6]. Therefore, resistance for tetracycline further curtails the list of cost effective first-line drugs for control of cholera.

All the* Vibrio cholerae *isolates in this study harboured class 1 integron and SXT constins. In this region,* qacE*Δ*1* and* sul1* genes provide resistance to ammonium compounds and sulphonamides, respectively. All the isolates were positive for* aadA2* and* dfrA1* genes, which confer resistance to streptomycin and trimethoprim, respectively. However, all the isolates were PCR negative for* blaP1 *involved in resistance for *β*-lactam antibiotics (Table 1). The SXT constin is a conjugative, self-transmissible, and integrative element that provides resistance to sulphamethoxazole, trimethoprim, chloramphenicol, and streptomycin. In this study, all isolates were susceptible to chloramphenicol and PCR negative for* floR *gene. In addition, the gene for streptomycin resistance (*strA*) was invariably present in all the isolates.

Integrons provide natural genetic engineering platforms that incorporate gene cassettes and convert them to functional genes by ensuring correct expression in bacteria. The present study showed that now* V. cholerae *outbreak strains carry the mobile antibiotic genetic elements and the horizontal transfer of these genes is the major cause of emergence of multidrug resistance strains. The commonly prescribed antibiotics in India for the treatment of diarrhea are fluoroquinolones (DNA damaging drugs) that induce horizontal transfer of SXT elements leading to inter- and intraspecies spread of multidrug resistance [34]. Therefore, use of such drugs should be closely monitored as most of the recent outbreak strains carry these elements [16, 19, 35].

MIC of tetracycline decreased eightfold in the presence of CCCP, indicating the role of efflux pumps in tetracycline resistance [36]. However, the MIC for nalidixic acid (32 *μ*g/mL) remained unaffected in the presence of CCCP indicating the involvement of some other mechanisms also for antibiotic resistance. Tetracycline resistance in Gram negative bacteria occurs due to the acquisition of genes encoding efflux pump and ribosomal protection proteins. Most of the efflux pumps belong to major facilitator superfamily (MFS), which export tetracycline from the cell and exchange a proton against the concentration gradient [37]. The isolates from this outbreak were PCR negative for the reported ribosomal protection protein Tet(M) in Vibrios (data not shown). Efflux mediated tetracycline resistance (*tet* genes) in Vibrios is mostly characterized in non-*V. cholerae* species [37]. Therefore, the detailed investigation of the mechanism is needed. In recent years, rise in antimicrobial resistance in clinical strains is a serious concern as it hampers outbreak control policies [10, 19, 35]. Thus, antimicrobial resistance can increase the outbreak size, duration, and case fatality rates. Continued monitoring of antimicrobial susceptibility as well as strain-tracking is important in adapting policies for cholera control at national and global levels.

## Figures and Tables

**Table 1:** Comparison of *V. cholerae *strains isolated during two cholera outbreaks in 2007 and 2010 from Rayagada, Odisha.

Year of isolation	*V. cholerae* strain	Gene traits	Antibiogram	Class 1 integrons	SXT	Antibiotic resistant gene	Reference
2007	O1 El Tor Ogawa	*ompW* ^+^, *ctxB* ^+^, *zot* ^+^, *rfbO1* ^+^, *tcp* ^+^, *ace* ^+^, *hlyA* ^+^, *ompU* ^+^, *rtx* ^+^, and *toxR* ^+^	Cot^R^, Nal^R^, Pol^R^, Spe^R^, Str^S^, Sul^R^, Tri^R^, Amp^R^, Tet^S^, Chl^S^, Ery^R^. Cip^S^, and Rif^S^	+	+	*aadA2* ^+^, *blaP1* ^−^, *dfrA1* ^+^, *floR* ^−^, and *strA* ^+^	[10, 19]

2010	O1 El Tor Ogawa	*ompW* ^+^, *ctxB* ^+^, *zot* ^+^, *rfbO1* ^+^, *tcp* ^+^, *ace* ^+^, *hlyA* ^+^, *ompU* ^+^, *rtx* ^+^, and *toxR* ^+^	Cot^R^, Nal^R^, Pol^R^, Spe^R^, Str^R^, Sul^R^, Tet^R^, Tri^R^, O/129^R^, Amp^S^, Chl^S^, Ery^S^. Cip^S^, and Rif^S^	+	+	*aadA2* ^+^, *blaP1* ^−^, *dfrA1* ^+^,* floR* ^−^, and *strA* ^+^	This study

Cot: co-trimoxazole, Nal: nalidixic acid, Pol: polymyxin B, Spe: spectinomycin, Str: streptomycin, Sul: sulfamethoxazole, Tet: tetracycline, Tri: trimethoprim, Amp: ampicillin, and Ery: erythromycin.

**Table 2:** Effect of CCCP on minimum inhibitory concentration (MIC) of various antibiotics on *V. cholerae* isolates.

Isolate	Tet	Nal	Str	Sul	Tri	Amp
	(*µ*g/mL)	(*µ*g/mL)	(*µ*g/mL)	(*µ*g/mL)	(*µ*g/mL)	(*µ*g/mL)
VCR4	16	32	≥1024	≥4096	≥1024	4
VCR4 + CCCP	2	32	≥1024	≥4096	256	4

## Nucleotide Sequence Accession Numbers

The GenBank accession numbers for the [ctxB] sequences of VCR4, VCR5, VCR6, VCR7, VCR8, and VCR9 are JX846970, JX846971, JX846972, JX846973, JX846974, and JX846975, respectively.
